# Breed and conformational predispositions for prolapsed nictitating membrane gland (PNMG) in dogs in the UK: A VetCompass study

**DOI:** 10.1371/journal.pone.0260538

**Published:** 2022-01-26

**Authors:** Dan G. O’Neill, Yahui Yin, Roser Tetas Pont, Dave C. Brodbelt, David B. Church, Camilla Pegram, Minna Mustikka

**Affiliations:** 1 Pathobiology and Population Sciences, The Royal Veterinary College, Hatfield, Herts, United Kingdom; 2 Clinical Science and Services, The Royal Veterinary College, Hatfield, Herts, United Kingdom; 3 Department of Equine and Small Animal Medicine, University of Helsinki, Helsinki, Finland; Universidade Federal de Minas Gerais, BRAZIL

## Abstract

**Background:**

Prolapsed nictitating membrane gland (PNMG) is the most common disorder of the third eyelid in dogs. However, the epidemiology of PNMG in the wider dog population remains understudied.

**Methods:**

Using de-identified clinical data from the VetCompass Programme, this cohort study aimed to report the prevalence, demographic and breed-related risk factors of PNMG in dogs attending UK primary care veterinary practices in 2016.

**Results:**

There were 1,802 PNMG cases identified from 905,543 dogs, yielding an annual prevalence of 0.20% (95% confidence interval (CI) 0.19–0.21). The median age at first diagnosis was 0.63 years (IQR 0.33–1.98, range 0.11–18.00). Dogs aged under 1 year had 10.82 times the odds (95% CI 9.17–12.76) compared with dogs aged from 2 to under 4 years. Neutered animals had higher odds than entire animals within both sexes. Breeds with the highest odds of PNMG compared with crossbred dogs included Neapolitan Mastiff (odds ratio (OR) 34.26, 95%CI 15.92–73.75), English Bulldog (OR 24.08, 95% CI 20.62–28.13), Cane Corso (OR 14.66, 95% CI 8.18–26.28), Lhasa Apso (OR 12.37, 95% CI 10.26–14.92) and American Cocker Spaniel (OR 11.57, 95% CI 5.59–23.96). Purebred dogs had 1.43 times the odds (95% CI 1.26–1.63) of PNMG compared with crossbreed dogs. Breeds with brachycephalic skull conformation had 6.71 times the odds (95%CI 5.89–7.64) compared with breeds with mesocephalic skull conformation. Insured dogs had 1.89 times the odds (95% CI 1.65–2.16) compared with uninsured dogs.

**Conclusions:**

This study reports the largest cohort of primary-care PNMG cases assembled to date. The results showing young age at diagnosis along with the breed, purebred and brachycephalic skull conformation predispositions suggest a hereditary involvement in PNMG development. These results may help to guide breeding strategies to reduce the prevalence of PNMG and improve welfare in predisposed individuals.

## Introduction

Prolapsed nictitating membrane gland (PNMG), often called “cherry eye”, describes a disorder where the nictitating membrane (accessory lacrimal) gland protrudes as a smooth or follicular pink mass from behind the leading edge of the third eyelid (nictitating membrane). The gland’s typical position is at the base of the third eyelid enveloping around the shaft of the T-shaped cartilage [1,2]. PNMG is the most common condition reported to affect the third eyelid in dogs, with a prevalence of 0.35% reported in a questionnaire survey on Kennel Club registered pedigree dogs in the UK [3]. Despite affected animals showing few signs of pain in the early stages [4], if left unattended or untreated, PNMG often results in chronic conditions such as keratoconjunctivitis sicca (KCS), inflammation, infection and trauma to the nictitating membrane gland itself, and potential secondary corneal trauma, all of which may result in discomfort or pain [1,2,4–6]. In a very small proportion of early and mild cases, topical antibiotic/corticosteroid therapy may be associated with control of the local inflammation and oedema of the conjunctiva, and a return of the nictitating membrane gland to its normal position [7]. However, in the majority of cases in which the gland is not surgically repositioned, long-term medical therapy will be required to control the inflammation and to improve the tear production [4,5,7].

The aqueous portion of the tear film in dogs comprises of contributions produced by the orbital lacrimal gland and the nictitating membrane gland that vary between individuals, with the nictitating membrane gland generally considered to produce approximately 30–60% [2,8,9]. Despite the observation of an initial compensatory increase of tear production in the remaining gland after surgical excision of either the nictitating membrane gland or the lacrimal gland, studies have indicated corneal micro-injuries and a decrease in quantitative tear production potentially resulting in KCS within a year after resection of the nictitating membrane gland [2,9]. Consequently, surgical excision of the prolapsed nictitating membrane gland is discouraged as a treatment option. Instead, surgical reposition of the gland is considered the gold standard therapy, with several surgical techniques described [2,5,10–15].

The precise aetiopathogenesis of PNMG is unknown. The condition is believed to result from abnormal laxity of the retinaculum formed by a portion of muscular fasciae of the orbit that attaches the lacrimal gland to the periorbita and third eyelid [1,8,16,17]. The conjunctival mucosa lining the posterior face of the third eyelid is populated with lymphoid tissue with the ductules of the nictitating membrane gland opening between the lymphoid follicles [2,18]. Although chronic hypertrophy of the nictitating membrane gland and hyperplasia of the conjunctiva-associated lymphoid tissue are often seen together with PNMG, it is yet to be determined whether these changes play a role as potential contributing factors or are merely consequences from the prolapse [7,19]. Deformities of the T-shaped cartilage of the third eyelid have been associated with PNMG as well as predisposing to re-prolapse after surgical correction of PNMG [1,19,20].

PNMG can be unilateral or bilateral, and is most often reported in dogs aged under two years [4,5,21]. Mazzucchelli et al [21] reported males to be overrepresented, while earlier studies failed to identify a sex predisposition [4,5,22]. Several breeds, including American Cocker Spaniel, Beagle, Boston Terrier, English Bulldog, French Bulldog, Great Dane, Lhasa Apso, Pekingese, Shar Pei, Shih Tzu, and Mastiff breeds have been suggested as predisposed to PNMG [2,3,5,13,19–21,23]. The brachycephalic skull conformation has also been suggested as a predisposing factor, although strong evidence of this is still outstanding [1,8,20,21,24]. However, deeper understanding of the epidemiology of PNMG in the wider dog population is limited because much of the currently available peer-reviewed literature has been based on studies with rather low sample sizes and often described populations from referral practice [10–12,15,20,21].

There is currently high interest in associations between breed and anatomical conformation with disease occurrence in order to identify possible etiopathogenetic pathways, contribute to improved breeding programmes and aid veterinary clinical management [25]. The current study aimed to generate epidemiological information on PNMG in the wider UK population of dogs under primary veterinary care. As well as reporting the estimated prevalence, the study aimed to explore demographic risk factors with a particular focus on associations with breed and conformation. The current study did not aim to report on clinical aspects of PNMG but instead left these as a topic for future study. Based on some prior evidence supporting a predisposition in dogs with brachycephalism [1,8,10–12,15,20,21,24], the current study hypothesized that brachycephalic dogs have an odds ratio of two or greater for PNMG compared with non-brachycephalic dogs.

## Materials and methods

The study population included all available dogs under primary veterinary care at clinics participating in the VetCompass Programme during 2016. Dogs under veterinary care were defined as those with either a) at least one electronic patient record (EPR) (VeNom diagnosis term [26], free-text clinical note, treatment or bodyweight) recorded during 2016 or b) at least one EPR recorded during both 2015 and 2017. VetCompass collates de-identified EPR data from primary-care veterinary practices in the UK for epidemiological research [27]. Data fields available to VetCompass researchers include a unique animal identifier along with species, breed, date of birth, sex, neuter status, insurance and bodyweight, and also clinical information from free-form text clinical notes, summary diagnosis terms [26] and treatment with relevant dates.

A cohort study design was used to estimate the one-year (2016) period prevalence of PNMG and to explore associations with demographic risk factors. Power calculations estimated that a study sample with at least 52,235 dogs was needed to estimate prevalence for a disorder that occurred in 0.35% of dogs with 0.05% acceptable margin of error at a 95% confidence level from a national UK population of 8 million dogs [28,29]. Ethics approval was obtained from the RVC Ethics and Welfare Committee (reference SR2018-1652).

The case definition for a PNMG case required evidence in the clinical records indicating the presence of prolapsed nictitating membrane gland or synonym (e.g., cherry eye, prolapsed nictitating membrane membrane gland, prolapsed third eyelid gland) in either eye at any date from 1st January 2016 to 31st December 2016. The clinical decision-making process was at the discretion of the attending veterinary surgeons. Case-finding involved initial screening of all 905,554 study dogs for candidate PNMG cases by searching the clinical free-text from 1st January 2016 to 31st December 2016 using the search terms: cherry, prol* nic*, prol* third, prol* 3rd, nict*, prol* gl*, prol* eyel*, prol* and TEL. Candidate cases were randomly ordered and the clinical notes of all 4,474 candidate animals were manually reviewed in detail to evaluate them for case inclusion.

Breed descriptive information entered by the participating practices was cleaned and mapped to a VetCompass breed list derived and extended from the VeNom Coding breed list that included both recognised purebred breeds and also designer breed terms [26]. A *purebred status* variable categorised all dogs of recognisable breeds as ‘purebred’, dogs with contrived names generated from two or more purebred breed terms as designers and dogs recorded as mixes of breeds but without a contrived name as ‘crossbred’ [30]. A *breed type* variable included individual pure breeds and designer hybrids represented by over 3000 dogs in the overall study population or with ≥ 5 PNMG cases, a grouped category of all remaining breed types and a grouping of general crossbred dogs. This approach was taken to facilitate statistical power for the individual breed analyses [31]. Breeds were further characterised by skull shape (dolichocephalic, mesocephalic, brachycephalic, uncategorised) and spaniel (spaniel, non-spaniel, uncategorised) status for analysis. A *Kennel Club breed group* variable classified breeds recognised by the UK Kennel Club into their relevant breed groups (Gundog, Hound, Pastoral, Terrier, Toy, Utility and Working) and all remaining types were classified as non-Kennel Club recognised [30].

Neuter and insurance status were defined by the final available EPR value. Adult bodyweight was defined as the mean of all bodyweight (kg) values recorded for each dog after reaching 18 months old and was categorised as: < 10.0, 10.0 to < 15.0, 15.0 to < 20.0, 20.0 to < 25.0, 25.0 to < 30.0, 30.0 to < 40.0 and ≥ 40.0. Mean adult bodyweight was generated for all breed/sex combinations with adult bodyweight available for at least 100 dogs in the overall study population and used to categorise individual dogs as “at or above the breed/sex mean”, “below the breed/sex mean” and “unspecified”. Age (years) was defined based on the first date for diagnosis of PNMG in the available clinical records for cases, and at December 31, 2016 (the final date in 2016 that these dogs were not a case) for non-cases. Age was categorised as: ≤ 1.0, 1.0 to < 2.0, 2.0 to < 4.0, 4.0 to < 6.0, 6.0 to < 8.0, 8.0 to < 10.0, 10.0 to < 12.0 and ≥ 12.0.

Following internal validity checking and data cleaning in Excel (Microsoft Office Excel 2013, Microsoft Corp.), analyses were conducted using Stata Version 16 (Stata Corporation).

One-year (2016) period prevalence with 95% confidence intervals (CI) was reported in dogs overall and in common breeds. The CI estimates were derived from standard errors based on approximation to the binomial distribution [32]. Risk factor analysis used binary logistic regression modelling to evaluate univariable associations between risk factors (*breed*, *skull shape*, *spaniel*, *purebred status*, *Kennel Club recognised breed*, *Kennel Club breed group*, *adult bodyweight*, *bodyweight relative to breed/sex mean*, *age*, *sex*, *neuter* and *insurance*) and PNMG during 2016. Because breed was a factor of primary interest for the study, variables that derived from the breed information and therefore were highly correlated with breed (*skull shape*, *spaniel*, *purebred status*, *Kennel Club recognised breed* and *Kennel Club breed group*) were excluded from initial breed multivariable modelling. Instead, each of these variables individually replaced the *breed* variable in the main final breed-focused model to evaluate their effects after taking account of the other variables. *Adult bodyweight* (a defining characteristic of individual breeds) replaced breed and *bodyweight relative to breed/sex mean* in the final breed-focused model. Risk factors with liberal associations in univariable modelling (*P* < 0.2) were taken forward for multivariable evaluation. Model development used manual backwards stepwise elimination. Clinic attended was evaluated as a random effect and pair-wise interaction effects were evaluated for the final model variables [33]. The area under the ROC curve and the Hosmer-Lemeshow test were used to evaluate the quality of the model fit and discrimination (non-random effect model) [33,34]. Statistical significance was set at *P* < 0.05.

## Results

### Prevalence

**Fig 1:**
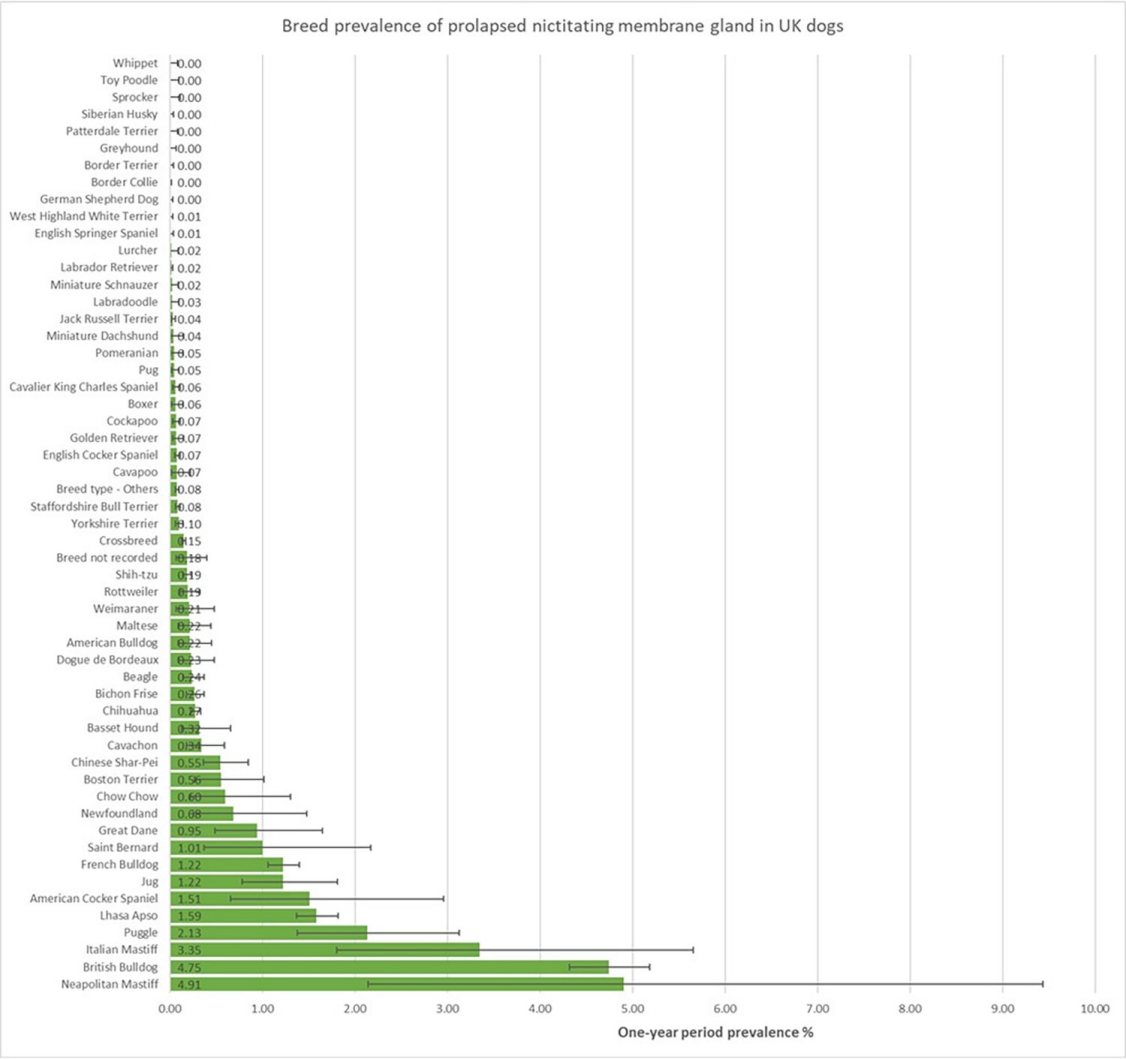
One-year (2016) period prevalence (percentage) of prolapsed nictitating membrane gland (PNMG) in dog breeds under primary veterinary care in the VetCompass™ Programme in the UK. The horizontal bars represent 95% confidence intervals.

The study included 905,553 dogs under veterinary care in 2016 at 887 veterinary clinics. From 4,474 candidate cases, there were 1,802 dogs with PNMG in 2016, giving an annual prevalence of 0.20% (95% CI: 0.19–0.21). The breeds with the highest one-year period prevalence for PNMG were Neapolitan Mastiff (4.91%, 95% confidence interval [CI] 2.14–9.44), English Bulldog (4.75%, 95% CI 4.32–5.19), Cane Corso (3.35%, 95% CI 1.80–5.66), Puggle (2.13%, 1.38–3.13) and Lhasa Apso (1.59%, 1.37–1.82) (Fig 1).

Of the PNMG cases with data available for that variable, 1,417 (78.90%) were purebred, 810 (45.15%) were female and 487 (27.15%) were neutered. Dogs with PNMG had a median adult bodyweight of 11.43kg (IQR: 7.70–22.90, range 2.42–78.50) and median age was 0.63 years (IQR: 0.33–1.98, range 0.11–18.00). The most common breed types among the PNMG cases were English Bulldog (n = 446, 24.75%), crossbreed (292, 16.20%), French Bulldog (200, 11.10%) and Lhasa Apso (199, 11.04%) (Table 1).

**Table 1:** Descriptive and univariable logistic regression results for breed as a risk factor for prolapsed nictitating membrane gland (PNMG) during 2016 in dogs under primary veterinary care in the VetCompass™ Programme in the UK. Column percentages shown in brackets.

Breed	Case No. (%)	Non-case No. (%)	Odds ratio	95% CI*	Category *P*-value	Variable *P*-value
Crossbreed	292 (0.15)	194,377 (99.85)	Base			< 0.001
Neapolitan Mastiff	8 (4.91)	155 (95.09)	34.36	16.73–70.57	< 0.001	
English Bulldog	446 (4.75)	8,953 (95.25)	33.16	28.57–38.49	< 0.001	
Cane Corso	13 (3.35)	375 (96.65)	23.08	13.12–40.59	< 0.001	
Puggle	25 (2.13)	1,148 (97.87)	14.50	9.60–21.9	< 0.001	
Lhasa Apso	199 (1.59)	12,350 (98.41)	10.73	8.95–12.86	< 0.001	
American Cocker Spaniel	8 (1.51)	521 (98.49)	10.22	5.04–20.74	< 0.001	
Jug	24 (1.22)	1,943 (98.78)	8.22	5.41–12.50	< 0.001	
French Bulldog	200 (1.22)	16,197 (98.78)	8.22	6.86–9.85	< 0.001	
Saint Bernard	6 (1.01)	591 (98.99)	6.76	3.00–15.23	< 0.001	
Great Dane	12 (0.95)	1,257 (99.05)	6.35	3.56–11.35	< 0.001	
Newfoundland	6 (0.68)	871 (99.32)	4.59	2.04–10.32	< 0.001	
Chow Chow	6 (0.6)	996 (99.4)	4.01	1.78–9.02	0.001	
Boston Terrier	10 (0.56)	1,789 (99.44)	3.72	1.98–7.00	< 0.001	
Chinese Shar-Pei	20 (0.55)	3,629 (99.45)	3.67	2.33–5.78	< 0.001	
Cavachon	12 (0.34)	3,523 (99.66)	2.27	1.27–4.04	0.006	
Basset Hound	7 (0.32)	2,169 (99.68)	2.15	1.01–4.55	0.046	
Chihuahua	101 (0.27)	36,693 (99.73)	1.83	1.46–2.30	< 0.001	
Bichon Frise	35 (0.26)	13,234 (99.74)	1.76	1.24–2.50	0.002	
Beagle	19 (0.24)	8,051 (99.76)	1.57	0.99–2.50	0.057	
Dogue de Bordeaux	7 (0.23)	3,025 (99.77)	1.54	0.73–3.26	0.259	
American Bulldog	7 (0.22)	3,217 (99.78)	1.45	0.68–3.07	0.333	
Maltese	7 (0.22)	3,241 (99.78)	1.44	0.68–3.05	0.343	
Weimaraner	5 (0.21)	2,410 (99.79)	1.38	0.57–3.35	0.475	
Rottweiler	14 (0.19)	7,271 (99.81)	1.28	0.75–2.19	0.365	
Shih-tzu	62 (0.19)	32,848 (99.81)	1.26	0.96–1.65	0.103	
Breed not recorded	6 (0.18)	3,280 (99.82)	1.22	0.54–2.73	0.633	
Yorkshire Terrier	27 (0.1)	28,153 (99.9)	0.64	0.43–0.95	0.026	
Staffordshire Bull Terrier	43 (0.08)	53,012 (99.92)	0.54	0.39–0.74	< 0.001	
Breed type—Others	64 (0.08)	84,425 (99.92)	0.50	0.38–0.66	< 0.001	
Cavapoo	3 (0.07)	4,032 (99.93)	0.50	0.16–1.55	0.226	
English Cocker Spaniel	24 (0.07)	33,051 (99.93)	0.48	0.32–0.73	0.001	
Golden Retriever	7 (0.07)	9,786 (99.93)	0.48	0.22–1.01	0.052	
Cockapoo	12 (0.07)	18,240 (99.93)	0.44	0.25–0.78	0.005	
Boxer	6 (0.06)	9,436 (99.94)	0.42	0.19–0.95	0.037	
Cavalier King Charles Spaniel	10 (0.06)	17,248 (99.94)	0.39	0.21–0.73	0.003	
Pug	8 (0.05)	16,206 (99.95)	0.33	0.16–0.66	0.002	
Pomeranian	3 (0.05)	6,218 (99.95)	0.32	0.10–1.00	0.05	
Miniature Dachshund	2 (0.04)	4,826 (99.96)	0.28	0.07–1.11	0.07	
Jack Russell Terrier	17 (0.04)	48,553 (99.96)	0.23	0.14–0.38	< 0.001	
Labradoodle	2 (0.03)	7,483 (99.97)	0.18	0.04–0.71	0.015	
Miniature Schnauzer	2 (0.02)	8,395 (99.98)	0.16	0.04–0.64	0.009	
Labrador Retriever	10 (0.02)	59,953 (99.98)	0.11	0.06–0.21	< 0.001	
Lurcher	1 (0.02)	6,021 (99.98)	0.11	0.02–0.79	0.028	
English Springer Spaniel	2 (0.01)	20,206 (99.99)	0.07	0.02–0.26	< 0.001	
West Highland White Terrier	1 (0.01)	18,877 (99.99)	0.04	0.00–0.25	0.001	
German Shepherd Dog	1 (0)	21,370 (100)	0.03	0.00–0.22	0.001	
Border Collie	0 (0)	24,388 (100)	~			
Border Terrier	0 (0)	9,651 (100)	~			
Greyhound	0 (0)	5,456 (100)	~			
Patterdale Terrier	0 (0)	4,455 (100)	~			
Siberian Husky	0 (0)	8,388 (100)	~			
Sprocker	0 (0)	3,338 (100)	~			
Toy Poodle	0 (0)	3,774 (100)	~			
Whippet	0 (0)	4,686 (100)	~			

*CI confidence interval.

Of the dogs that were not PNMG cases with data available on the variable, 653,485 (72.57%) were purebred and 430,898 (47.90%) were female, 407,478 (45.30%) were neutered. The median adult bodyweight for non-cases was 13.97 kg (IQR: 8.20–25.02, range 0.72–97.20) and the median age was 4.45 years (IQR: 1.88–8.09, range 0.00–20.97). The most common breeds among the non-case dogs were crossbred (n = 194,377, 21.51%), Labrador Retriever (59,593, 6.63%), Staffordshire Bull Terrier (52,012, 5.87%) and Jack Russell Terrier (48,553, 5.37%) (Table 1).

### Risk factors

**Fig 2:**
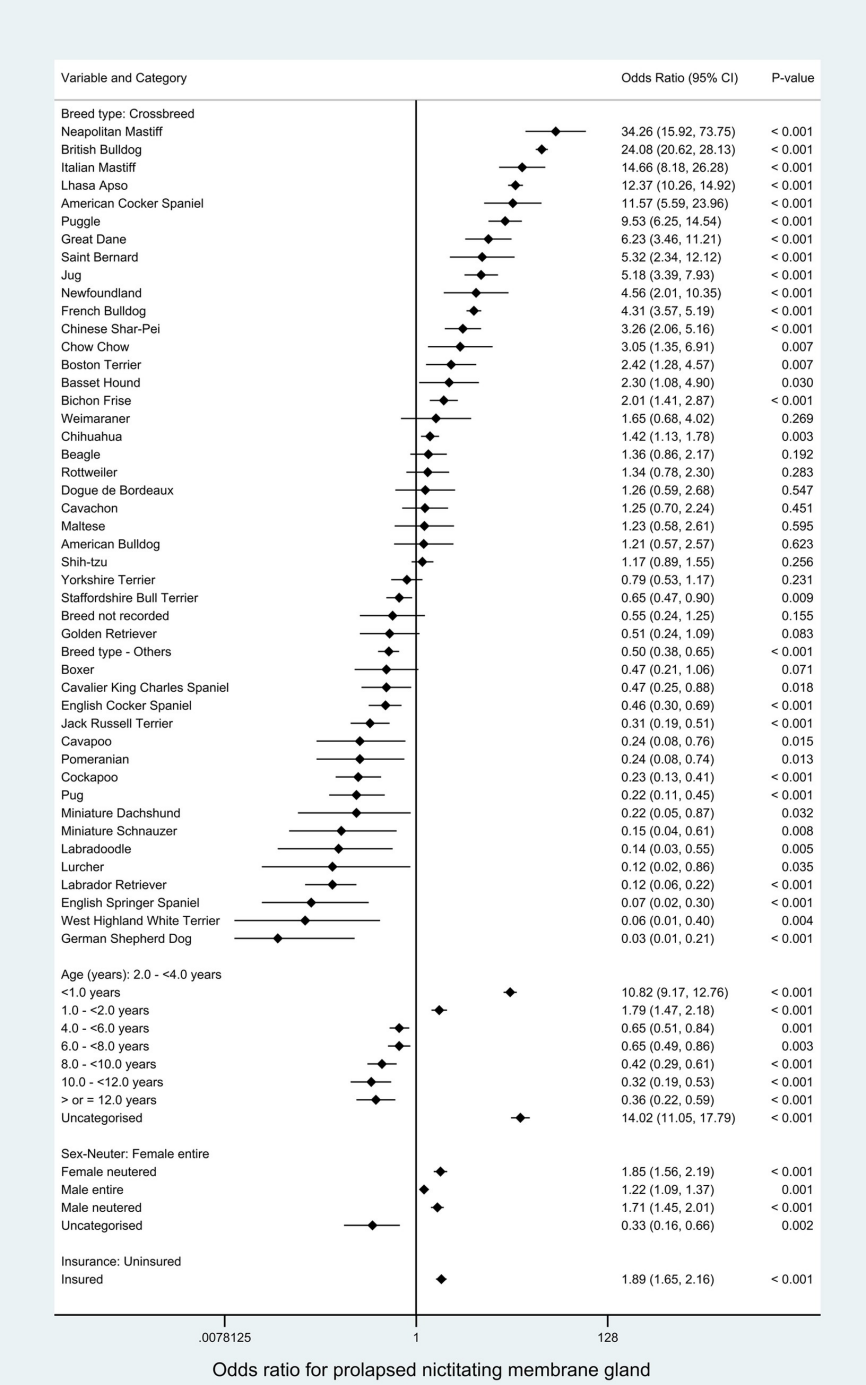
Final breed-focused mixed effects multivariable logistic regression model for risk factors associated with prolapsed nictitating membrane gland (PNMG) in dogs under primary veterinary care in the VetCompass Programme in the UK. Clinic attended was included as a random effect. *CI confidence interval.

All variables were liberally associated with PNMG in univariable logistic regression modelling and were therefore evaluated using multivariable logistic regression modelling (Tables 1–3). The final main breed-focused multivariable model retained four risk factors: *breed*, *age*, *sex-neuter* and *insurance* (Fig 2). No biologically significant interactions were identified. The final model was improved by inclusion of the clinic attended as a random effect (rho: 0.04 indicating that 4% of the variability was accounted for by the clinic attended, *P* < 0.001). The final model showed acceptable model-fit (Hosmer-Lemeshow test statistic: *P* = 0.232) and acceptable discrimination (area under the ROC curve: 0.890).

**Table 2:** Descriptive and univariable logistic regression results for breed-derived risk factors for prolapsed nictitating membrane gland (PNMG) during 2016 in dogs under primary veterinary care in the VetCompass™ Programme in the UK. Column percentages shown in brackets.

Variable	Category	Case No. (%)	Non-case No. (%)	Odds ratio	95% CI*	Category *P*-value	Variable *P*-value
Purebred status	Crossbred	292 (0.15)	194,377 (99,85)	Base			< 0.001
	Designer	87 (0.17)	52,599 (99.83)	1.10	0.87–1.40	0.431	
	Purebred	1,417 (0.22)	653,485 (99.78)	1.44	1.27–1.64	< 0.001	
Kennel Club Recognised Breed	Not recognised	413 (0.16)	261,942 (99.84)	Base			< 0.001
	Recognised	1,383 (0.22)	638,519 (99.78)	1.37	1.23–1.53	< 0.001	
Kennel Club Breed Group	Not Kennel Club recognised breed	413 (0.16)	261,942 (99.84)	Base			< 0.001
	Terrier	72 (0.05)	145,849 (99.95)	0.31	0.24–0.40	< 0.001	
	Gundog	61 (0.04)	135,612 (99.96)	0.29	0.22–0.37	< 0.001	
	Working	70 (0.18)	39,147 (99.82)	1.13	0.88–1.46	0.331	
	Pastoral	2 (0.00)	52,980 (100.00)	0.02	0.01–0.10	< 0.001	
	Utility	939 (0.91)	101,725 (99.09)	5.85	5.21–6.57	< 0.001	
	Hound	39 (0.12)	31,378 (99.88)	0.79	0.57–1.09	0.156	
	Toy	200 (0.15)	131,828 (99.85)	0.96	0.81–1.14	0.655	
Skull conformation	Mesocephalic	290 (0.07)	417,354 (99.93)	Base			< 0.001
	Brachycephalic	1,094 (0.65)	166,339 (99.35)	9.47	8.31–10.77	< 0.001	
	Dolichocephalic	33 (0.05)	69,792 (99.95)	0.68	0.47–0.98	0.036	
	Uncategorised	385 (0.15)	250,256 (99.85)	2.21	1.90–2.58	< 0.001	
Spaniel	Non spaniel-type	1,364 (0.24)	576,588 (99.76)	Base			< 0.001
	Spaniel-type	53 (0.07)	76,897 (99.93)	0.29	0.22–0.38	< 0.001	
	Uncategorised	385 (0.15)	250,256 (99.85)	0.65	0.58–0.73	< 0.001	

*CI confidence interval.

**Table 3:** Descriptive and univariable logistic regression results for non-breed-related demographic risk factors evaluated for prolapsed nictitating membrane gland (PNMG) during 2016 in dogs under primary veterinary care in the VetCompass™ Programme in the UK. Column percentages shown in brackets.

Variable	Category	Case No. (%)	Non-case No. (%)	Odds ratio	95% CI*	Category *P*-value	Variable *P*-value
Adult (> 18 months) bodyweight (kg)	< 10.0	391 (0.18)	212,961 (99.82)	Base			< 0.001
	10.0 - < 15.0	172 (0.17)	98,216 (99.83)	0.95	0.80–1.14	0.606	
	15.0 - < 20.0	74 (0.11)	69,317 (99.89)	0.58	0.45–0.75	< 0.001	
	20.0 - < 25.0	120 (0.19)	63,787 (99.81)	1.03	0.83–1.26	0.816	
	25.0 - < 30.0	77 (0.14)	53,697 (99.86)	0.78	0.61–1.00	0.048	
	30.0 - < 40.0	58 (0.08)	69,874 (99.92)	0.45	0.34–0.60	< 0.001	
	≥ 40.0	44 (0.17)	26,213 (99.83)	0.91	0.70–1.25	0.573	
	Uncategorised	866 (0.28)	309,676 (99.72)	1.52	1.35–1.72	< 0.001	
Bodyweight relative to breed mean	Lower	520 (0.16)	316,829 (99.84)	Base			< 0.001
	Equal/Higher	413 (0.15)	275,100 (99.85)	0.91	0.80–1.04	0.177	
	Uncategorised	869 (0.28)	311,812 (99.72)	1.70	1.52–1.89	< 0.001	
Age (years)	< 1.0	1,021 (0.99)	102,306 (99.01)	9.41	8.05–10.99	< 0.001	< 0.001
	1.0 - < 2.0	234 (0.18)	130,314 (99.82)	1.69	1.40–2.05	< 0.001	
	2.0 - < 4.0	189 (0.11)	178,170 (99.89)	Base			
	4.0 - < 6.0	93 (0.07)	139,588 (99.93)	0.63	0.49–0.81	< 0.001	
	6.0 - < 8.0	68 (0.06)	113,449 (99.94)	0.57	0.43–0.75	< 0.001	
	8.0 - < 10.0	33 (0.04)	90,870 (99.96)	0.34	0.24–0.50	< 0.001	
	10.0 - < 12.0	16 (0.02)	66,497 (99.98)	0.23	0.14–0.38	< 0.001	
	≥ 12.0	18 (0.03)	70,143 (99.97)	0.24	0.15–0.39	< 0.001	
	Uncategorised	130 (1.04)	12,404 (98.96)	9.88	7.90–12.36	< 0.001	
Sex	Female	810 (0.19)	430,898 (99.81)	Base			0.065
	Male	984 (0.21)	468,622 (99.79)	1.12	1.02–1.23	0.020	
	Uncategorised	8 (0.19)	4,221 (99.81)	1.01	0.50–2.02	0.982	
Neuter	Entire	1,307 (0.26)	492,044 (99.74)	Base			< 0.001
	Neutered	487 (0.12)	407,478 (99.88)	0.45	0.41–0.50	< 0.001	
	Uncategorised	8 (0.19)	4,219 (99.81)	0.71	0.36–1.43	0.342	
Insurance	Non-insured	1,475 (0.19)	786,500 (99.81)	Base			< 0.001
	Insured	327 (0.28)	117,241 (99.72)	1.49	1.32–1.68	< 0.001	

*CI confidence interval.

After accounting for the effects of the other variables evaluated, 17 breeds showed increased odds of PNMG compared with crossbred dogs. The breeds with the highest odds included Neapolitan Mastiff (odds ratio [OR] 34.26, 95% CI 15.92–73.75, P < 0.001) and English Bulldog (OR 24.08, 95% CI 20.62–28.13, P < 0.001). Sixteen breeds showed reduced odds of PNMG compared with crossbreds. Dogs aged under 1 year had 10.82 (95% CI 9.17–12.76, P < 0.001) times the odds compared with dogs aged from 2 to under 4 years. Sex per se was not associated with the odds of PNMG but neutered animals had higher odds than entire animals within both sexes. Insured dogs had 1.89 (95% CI 1.65–2.16, *P* < 0.001) times the odds of PNMG compared with uninsured dogs (Fig 2).

**Fig 3:**
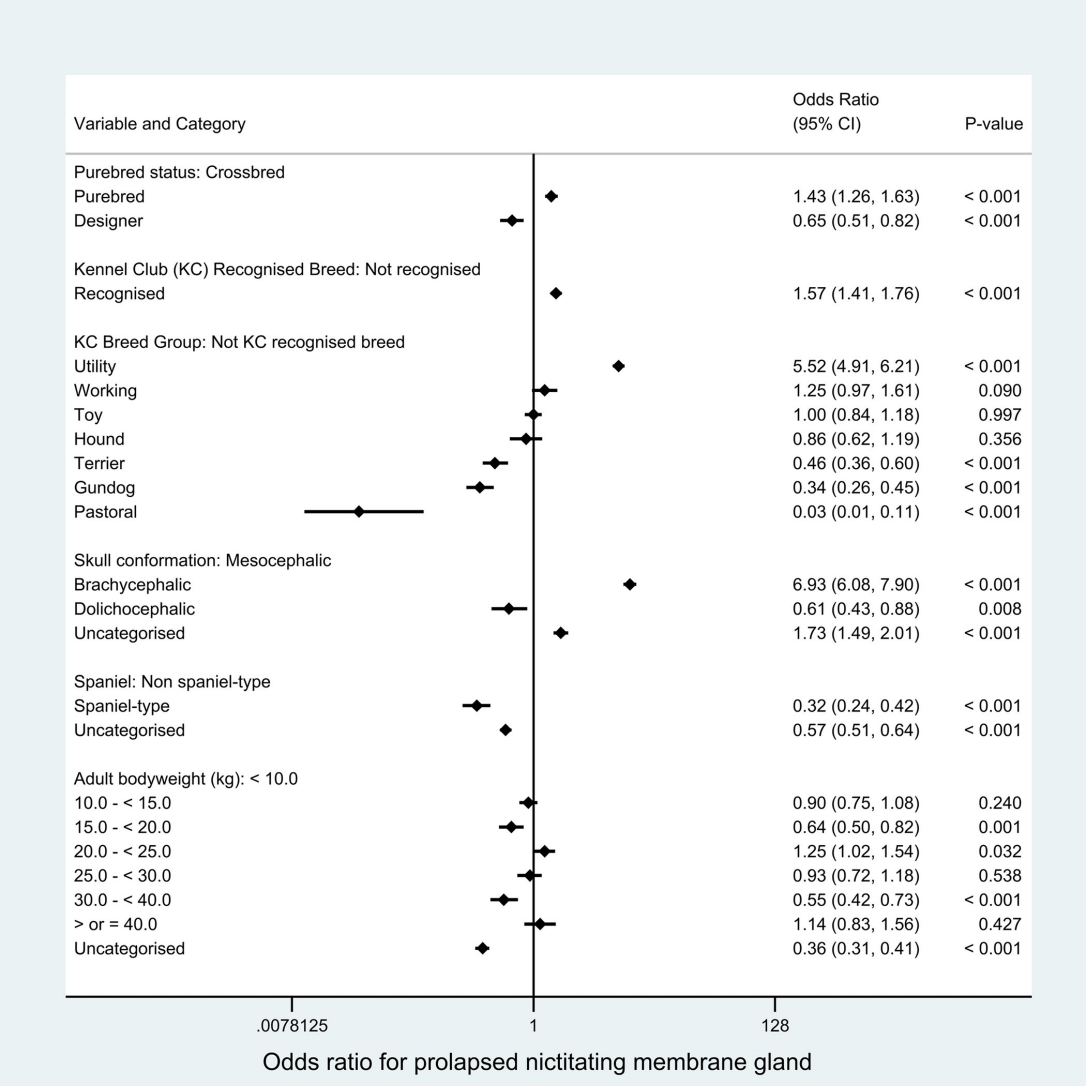
Results for risk factors that directly replaced the breed variable in the final breed-focused mixed effects multivariable logistic regression model (along with age, sex/neuter and insurance status). Adult (> 18 months) bodyweight (kg) replaced the breed and bodyweight relative to breed mean variables in the final breed-focused mixed effects multivariable logistic regression model. These results report associations between these risk factors and prolapsed nictitating membrane gland (PNMG) in dogs under primary veterinary care in the VetCompass™ Programme in the UK. Clinic attended was included as a random effect. *CI confidence interval.

As described in the methods, breed-derived variables were introduced individually to replace *breed type* in the final breed-focused model. Compared with crossbred dogs, purebred dogs had increased odds (OR 1.43, 95% CI 1.26–1.63, P < 0.001) while designer types had reduced odds (OR 0.65, 95% CI 0.51–0.82, *P* < 0.001) of PNMG. Among the Kennel Club breed groups, Utility showed higher odds while Pastoral, Gundog and Terrier groups showed lower odds of PNMG compared with breeds that are not recognized by the Kennel Club. Compared with breeds with mesocephalic skull conformation, breeds with brachycephalic skull conformation (OR 6.93, 95% CI 6.08–7.90, P < 0.001) had increased odds while breeds with dolichocephalic (OR 0.61, 95% CI 0.43–0.88, P = 0.008) skull conformations had reduced odds of PNMG. Spaniel types had 0.32 times the odds (95% CI 0.24–0.42, P < 0.001) of PNMG compared with non-spaniel types. Adult bodyweight was variably associated with the odds of PNMG across the bodyweight categories (Fig 3).

## Discussion

This study explores the occurrence of PNMG in a sample of over 900,000 dogs under UK first opinion veterinary care in 2016. The large size of the study population offered opportunities to report and compare the occurrence of PNMG within and between many breeds while still retaining good precision in the results. The breed and conformational predispositions identified here support a hereditary basis for PNMG. Several other important and novel predisposing factors are also reported. The strong association shown with brachycephalism in combination with predispositions in certain specific breeds suggest that the aetiopathogenesis is linked to characteristics of skull conformation in certain types of dogs. This information can act as a hypothesis generator for future research aimed specifically at elucidating the aetiopathogenesis of PNMG and may assist to identify new therapeutic options.

The current study reported an annual prevalence of 0.20% for PNMG from an underlying population of close to a million dogs under primary veterinary care. An earlier survey of owners of pedigree dogs registered with The Kennel Club reported a higher prevalence of 0.35% but that higher result might be expected because that study was limited to a predisposed pedigree subset of the wider dog population and that study also reported lifetime prevalence whereas the current study reported one-year period prevalence [3]. Awareness of the impact on results from differences in study design highlights the importance of taking context into account when interpreting and generalising from the results of individual studies [35,36]. Reliable prevalence data is critical to evaluating and comparing the welfare impact of specific disorders. Welfare impact assessment requires information on prevalence, duration and severity [37]. The high prevalence values reported in the current study in relation to certain predisposed breeds suggest that PNMG contributes substantially to the overall disorder burden in these breeds and therefore should be considered for prioritisation within health schemes for these breeds [25].

Purebred dogs were 1.43 times more likely to be diagnosed with PNMG compared to crossbreed dogs in the current study. This finding is in accordance with previous studies reporting lower percentages of crossbreed dogs with PNMG in referral population settings [5,11,12,15,20]. A purebred predisposition provides further support for a presumed hereditary basis of the disorder. Amongst the PNMG cases, English Bulldogs (24.75% of the cases) and crossbreed (16.20%) were the two most common breeds, but this finding should not be over-interpreted because it is highly influenced by the relative popularity of these breeds in the UK. Moreover, crossbreed was the most common type of dog (21.51%) amongst non-affected cases. Breed popularity varies widely between countries across the world and therefore the prevalence of PNMG in any one country will largely reflect the relative popularity and health of predisposed and protected breeds in that country [38–40]. Geographic isolation, founder effects and variation in breeding practices may also lead to internationally differing conformations and genetics between ostensibly the same breed, and these differences could also support differing national predispositions to PNMG within breeds [41]. Similarly, the breed popularity and the customer base around the referral practices used as the data source for previous studies are likely to have had a strong influence on the breed results from previous PNMG studies [5,11,12,15,20,21].

When assessing the relative risks of PNMG across various breeds, the current study used multivariable methods to take account of possible confounding variables including age, sex, neuter and insurance in order to give more nuanced and reliable results [33]. After accounting for the confounding effects of the other demographic variables, breed was identified as a very strong predictor of PNMG dogs, with 17 breeds showing increased odds and 16 breeds showing reduced odds compared with crossbred dogs. All of the predisposed breeds, except for Puggle, have previously been included among lists of commonly affected breeds [2,3,5,13,19–21,23] and are recognised as breeds with “presumed inherited” PNMG by the European College of Veterinary Ophthalmologists Hereditary Eye Disease (ECVO-HED) Committee [42]. The breeds with the highest odds included Neapolitan Mastiff (OR 34.26), English Bulldog (OR 24.08), Cane Corso (OR 14.66), Lhasa Apso (OR 12.37) and American Cocker Spaniel (OR 11.57). Our findings of specific breeds with remarkably high odds for PNMG further supports a strong genetic predisposition to PNMG in dogs. In line with our findings, a study of animals presented at dog shows and for referral veterinary care in Italy identified high prevalence of PNMG in Neapolitan Mastiff and Cane Corso, while a third Italian breed, Maremma Sheepdog, showed relatively low prevalence of PNMG [23]. This finding further supports a genetic predisposition for PNMG, because Neapolitan Mastiff and Cane Corso are genetically closely related, while Maremma Sheepdog is genetically more distant from both of them [43]. However, the results of the current study in relation to the various types of spaniels raise some questions about the strength of genetic associations within these breeds. The American Cocker Spaniel is widely cited as a commonly affected breed for PNMG in the previous literature [2,3,5,13,19–21,23]. The current study supports a strong predisposition by reporting the American Cocker Spaniel with over 11 times the odds of PNMG compared to crossbreds. However, the current study also reports strong protective effects in the English Springer Spaniel (OR 0.07) and English Cocker Spaniel (OR 0.46), despite all of these spaniel types being reportedly phylogenetically close [43]. A more brachycephalic conformation in the American Cocker Spaniel compared with the other spaniel breeds may partially explain the differing risk of PNMG [30].

Designer breeds (or types) represent specified crosses between differing parental purebred breeds [44]. Although designer breeds as an overall group had reduced odds of PNMG overall (OR 0.65) compared to the general crossbred group, not all individual designer breeds were protected to PNMG. Some designer breeds, such as the Puggle (Pug and Beagle hybrid), showed high odds for PNMG (OR 9.53) despite no predisposition being shown in the Beagle (OR 1.36) and the Pug showing active protection (OR 0.22). A similar phenomenon was shown in the Jug (Jack Russell Terrier and Pug hybrid) that showed an odds ratio of 5.18 despite both parental breeds showing active protection to PNMG (Pug OR 0.22, Jack Russell Terrier OR 0.31). The discovery of designer hybrids with high risk for PNMG despite being crossed of parental breeds with low risk emphasizes the complexity of the inheritance of PNMG and highlights that the health status of the parent breeds may be a poor predictor of the health status of subsequent hybrids [45]. These findings also provide some evidence against the theory that hybrid vigour from planned crosses will result in substantially improved health status [46].

Based on previous reports that brachycephalism was associated with PNMG in dogs [1,8,20,21,24], the current study explicitly explored brachycephalism as a study hypothesis. The results showed overwhelming support for the hypothesis of predisposition to PNMG in brachycephalic breeds by reporting an odds ratio of 6.93 for PNMG in brachycephalic breeds compared with non-brachycephalic breeds. Crowding of the inferior orbital space in brachycephalic breeds has been suggested as a factor contributing to PNMG [8,47]. The zygomatic salivary gland is typically located in the periorbital area medial to the zygomatic arch and lateral to the medial pterygoid muscle [17,48]. Atypical location of the zygomatic salivary gland directly beneath the skin ventral to the zygomatic arch, lateroventral to the medial pterygoid muscle and rostroventral to the masseter muscle, has been described in small breed and brachycephalic dogs [48]. The nictitating membrane gland is presumably remarkably more easily prolapsed outside of the orbit than the zygomatic gland, due to the inclined location and much smaller size than the zygomatic salivary gland [49,50]. Therefore, PNMG may be related to the anatomical qualities in the skull conformation resulting from crowding of the orbital space in brachycephalic breeds. Future work, potentially aided by diagnostic imaging, is required on investigating the relative size and position of tissues in orbits affected by PNMG.

Despite a clear predisposition in brachycephalic breeds overall, a high level of risk variation was seen between the common brachycephalic breeds. The breeds with the highest odds for PNMG, Neapolitan Mastiff (OR 34.26), English Bulldog (OR 24.08), Cane Corso (OR 14.66) and American Cocker Spaniel (OR 11.57), all show brachycephalic skull conformations and have close phylogenetic relationships [43,51]. However, other common brachycephalic breeds, such as the Shih-Tzu (OR 1.17) and Boxer (OR 0.47), did not show any predisposition and the Pug (OR 0.22) was a highly protected breed. These findings suggest that, although the brachycephalic skull conformation is clearly associated with PNMG, there are clearly many other factors in play at a breed level. Safe inference from the results of epidemiological studies should focus on interpreting the health status of dogs at a breed level of abstraction as well as at higher levels of abstraction, such as based on skull conformation [51].

The precise aetiopathogenesis of PNMG remains unclear. All five breeds with the highest odds of PNMG in the current study (Neapolitan Mastiff, English Bulldog, Cane Corso, Lhasa Apso and American Cocker Spaniel) are described with excessively long eyelids in relation to the size of the globe and orbit (macroblepharon) and consequently to show excessively large palpebral fissures (euryblepharon/macropalpebral fissure) as a presumed inherited disorder [42]. On the other hand, some other breeds commonly displaying euryblepharon/macropalpebral fissure such as Pug and Shih Tzu were protected from PNMG in the current study [42]. While the euryblepharon/macropalpebral fissure as such does not seem to predispose to PNMG, macropalpebral fissure and macroblepharon are often seen in conjunction with laxity of the lateral canthal ligament and supportive structures [42,52]. Moreover, PNMG with concomitant eyelid anomaly has been reported as significantly more common in giant breeds [53]. These findings could support a role for excessive periocular tissue laxity and thus lack of support for the nictitating membrane in the etiopathogenesis of PNMG. The observations regarding a potential link between eyelid disorders and predisposition for PNMG highlight the importance of overall functional and healthy eyelid conformation as one of the key objectives in breeding strategies aiming to reduce the prevalence of PNMG [25].

The median age in the current study at first diagnosis of PNMG across all dog breeds was 0.63 years. Given that the breed predispositions in the current study suggest a strong inherited tendency towards PNMG, the low median age of diagnosis may offer an advantage for breeders aiming to select away from this disorder by removing affected animals from breeding pools. The odds for PNMG diagnosis were substantially reduced in dogs at all ages over two years, so avoiding breeding until this age could provide a relatively high level of confidence of the individual animal’s potential for PNMG.

Approximately 39.6% of the dogs in the UK have been reported to undergo neutering within the first year of life [54]. There was some evidence of increased odds for PNMG in the neutered dogs compared to entire ones, but this may reflect the effects of reverse causality rather than any direct causality. The neuter status used in the study was the value recorded at the final available clinical record. Animals diagnosed with PNMG may be more likely to undergo neutering than non-affected dogs because affected animals might be considered as less suitable for breeding [42]. This shows the benefits from deeper understanding of data sources before reaching conclusions and generating inference from research findings.

Insured dogs had 1.5 times the odds of a recorded diagnosis during 2016 with PNMG. Given that it would be anticipated that all presented cases of PNMG would receive a veterinary diagnosis, this suggests a considerable level of under-presentation of PNMG for veterinary care from the uninsured subset of the wider dog population. This under-reporting of the true cases in the wider dog population should be taken into account when interpreting the results presented here that reflect the apparent prevalence of diagnosed cases rather than the true prevalence that includes both diagnosed and undiagnosed cases [55]. Insurance has been similarly associated with increased diagnostic rates for other disorders in dogs including corneal ulceration (x1.6) [56], patellar luxation (x1.9) [57], urinary incontinence (x1.6) [58] and lipoma (x1.8) [59]. Higher diagnosis within insured dogs may result from lower thresholds of concern before owners of insured dogs seek veterinary care and higher levels of care provided by veterinary professionals [60]. These findings suggest that pet insurance results in a positive impact on the welfare of the dog, whereas owners of uninsured dogs may not always seek veterinary care when needed.

This study had some limitations in addition to those that have been previously reported in the application of primary care veterinary clinical records for research [51,61]. The information on neuter related to the status at the final available record and therefore may have been different to the status at the time of diagnosis of PNMG. The date of diagnosis describes the date of first formal veterinary diagnosis but the condition could have been present in each dog for varying lengths of preceding time [4]. While access to the primary care world of clinical data has been discussed above as a major strength of this study in terms of generalisability of the results, this data resource also behaves as a limitation in certain other respects such as deeper exploration of potential aetiopathogenesis. Time constraints typically enforced upon primary care consultations may preclude primary care veterinarians from assessing and recording the entire spectrum of issues relating to ocular health in detail during initial presentation of these PNMG cases.

## Conclusions

PNMG is a relatively common diagnosis in dogs overall and more so within certain predisposed breeds, with over one in 25 dogs from breeds such as the Neapolitan Mastiff and English Bulldog affected annually. Brachycephalic skull conformation was also associated with almost seven times higher odds ratio of PNMG. These findings suggest inherited and conformational bases for the condition. However, strong predispositions in several designer breeds, despite being crossed from parental breeds with low risk of PNMG, highlights the complexity of inheritance of PNMG. The disorder was much more common in young dogs suggesting that delaying breeding decisions until later may assist in breeding away from PNMG.

## Abbreviations

CI: confidence interval
EPR: electronic patient record
IQR: interquartile range
IRR: incidence risk ratio
KC: The Kennel Club
KCS: keratoconjunctivitis sicca
OR: odds ratio
